# Supplementary material for: Identification of electroporation sites in the complex lipid organization of the plasma membrane
Source: eLife. 2022 Feb 23;11:e74773. doi: 10.7554/eLife.74773 (PMC8912918; doi:10.7554/eLife.74773)

Histograms for APM. Histograms showing the values of tested features in nonporated (empty bars) and porated (blue bars) locations. The purple and yellow solid lines show kernel smoothing density estimates. The title above each group of graphs indicates the dataset for which the histograms are shown.

APM - depolarization - inner leaflet

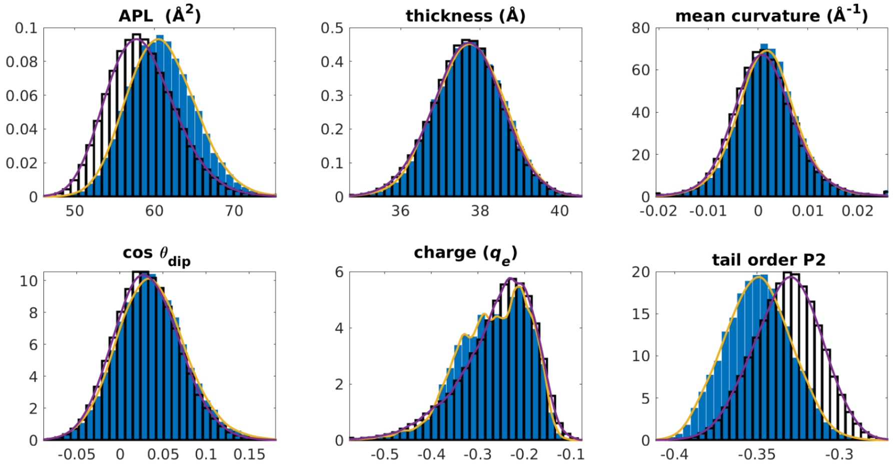

APM - depolarization - outer leaflet

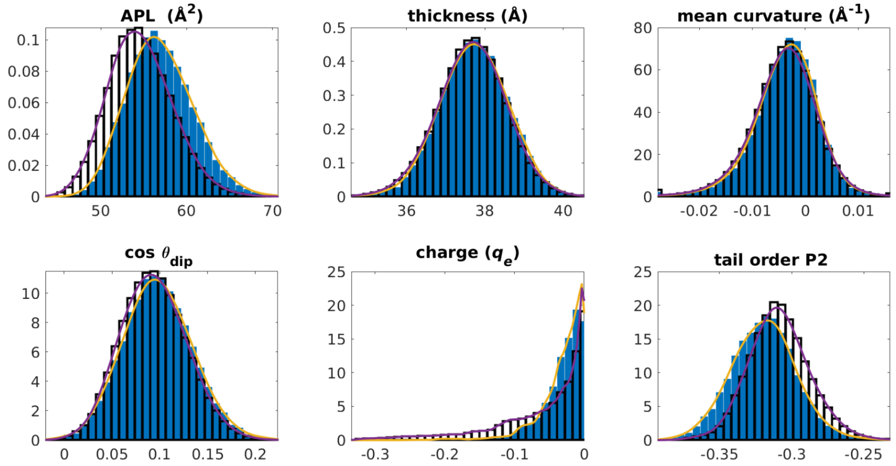

APM - hyperpolarization - inner leaflet

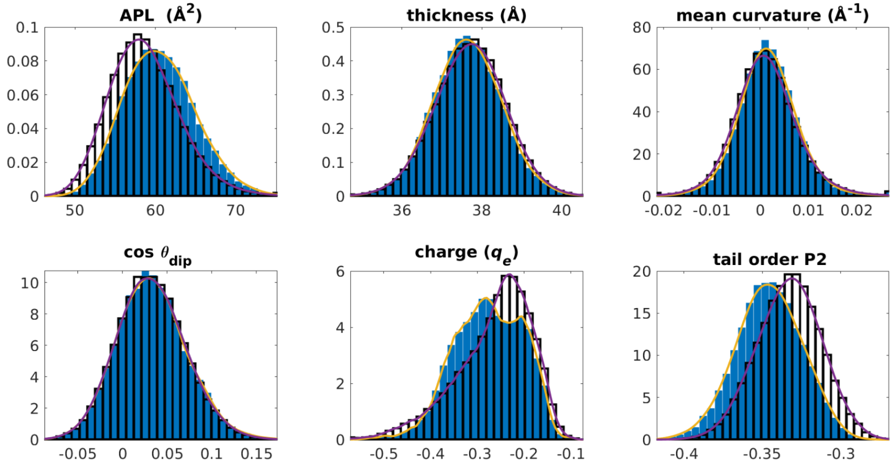

APM - hyperpolarization - outer leaflet

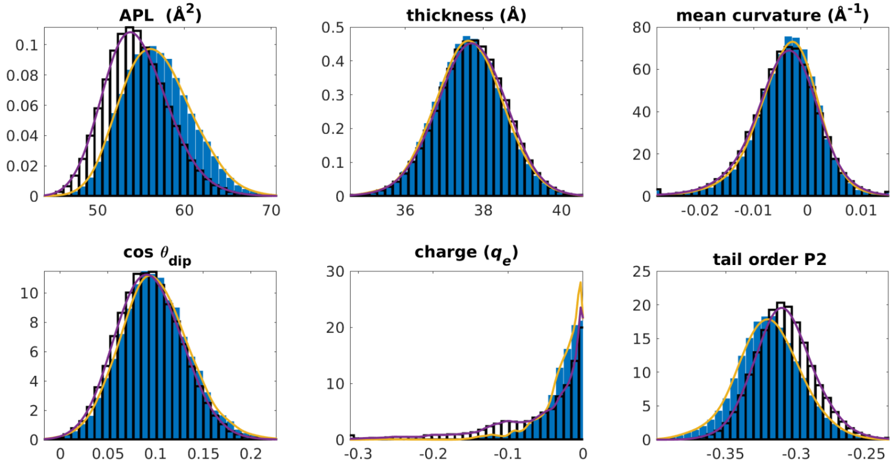

Histograms for APM - continued.

APM - depolarization - inner leaflet

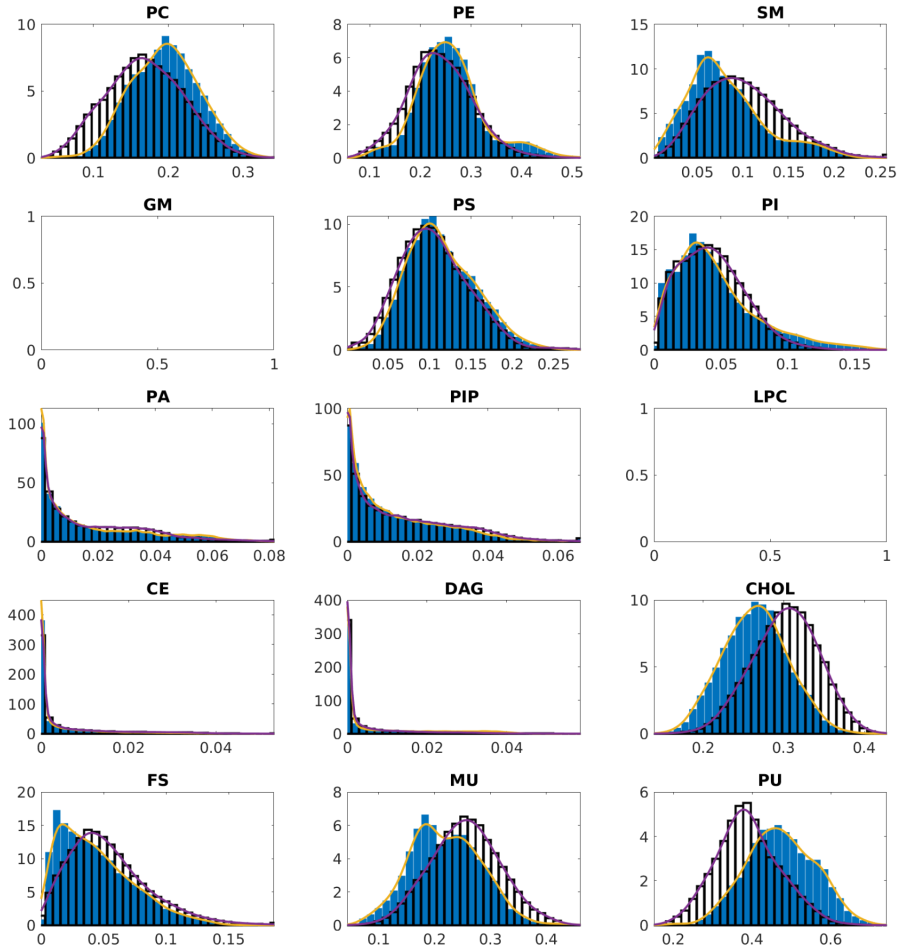

APM - depolarization - outer leaflet

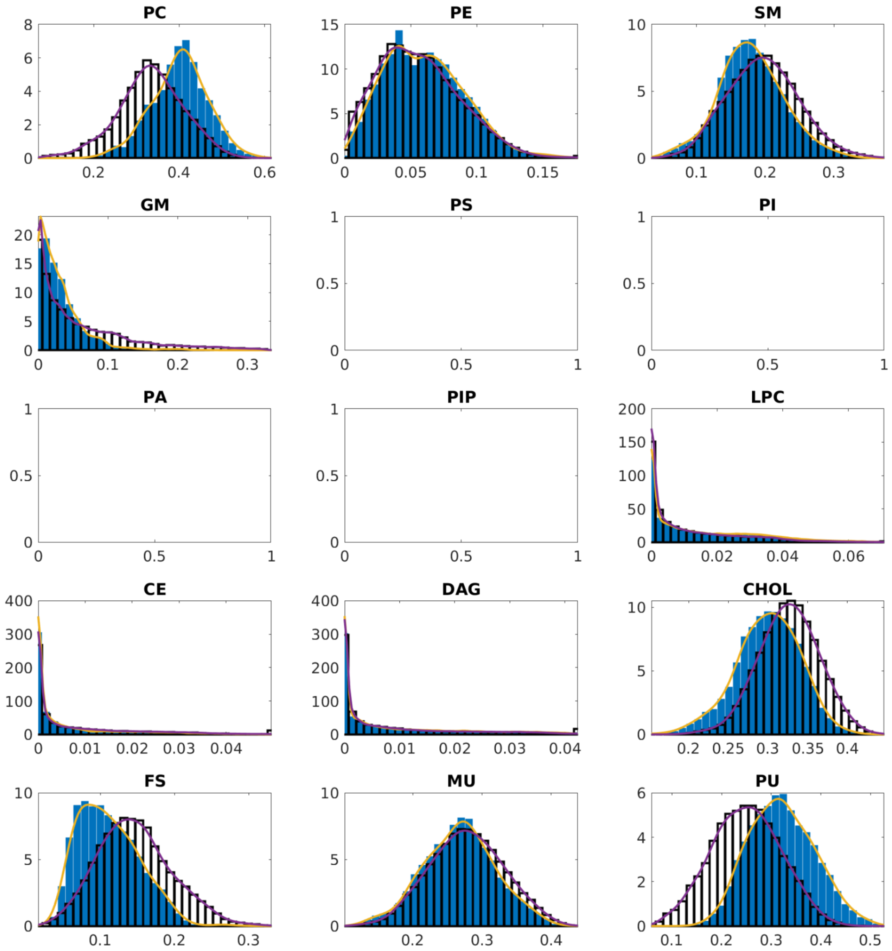

Histograms for APM - continued.

APM - hyperpolarization - inner leaflet

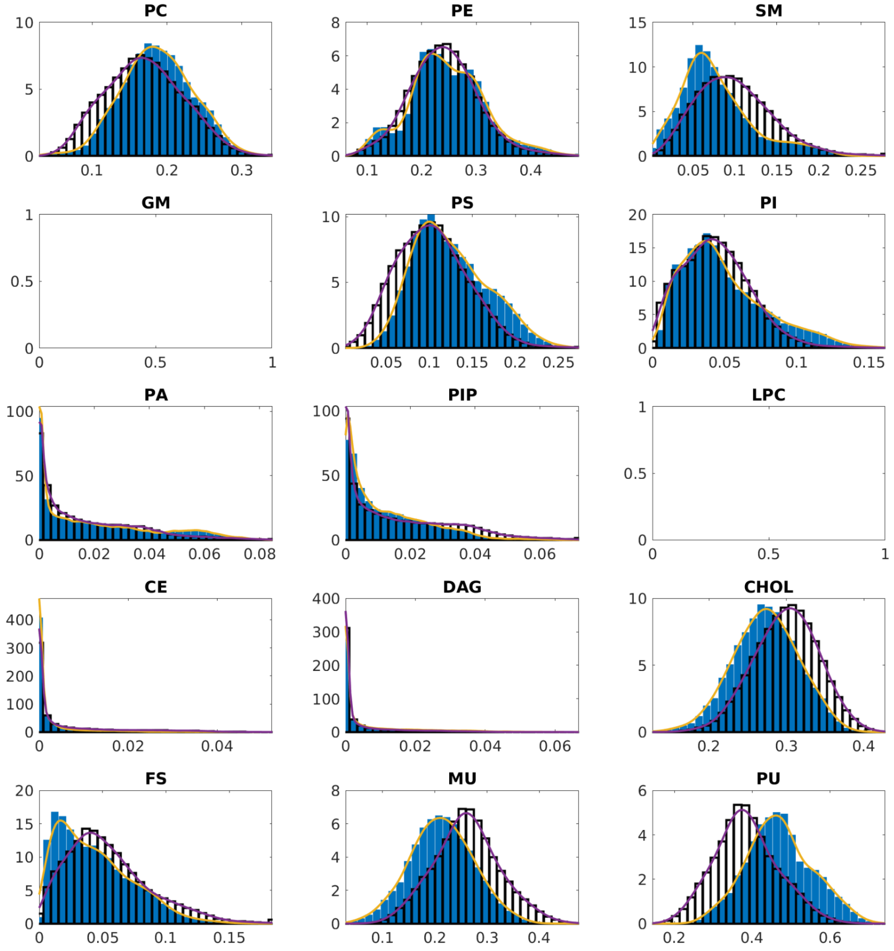

APM - hyperpolarization - outer leaflet

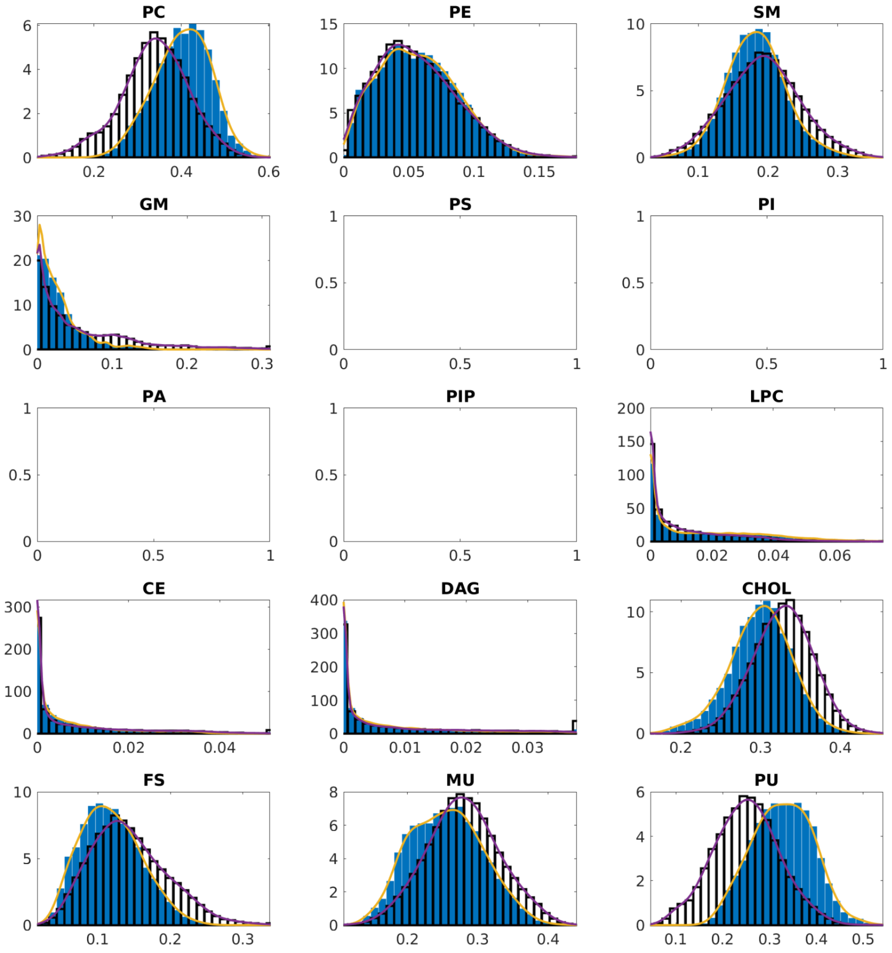

Histograms for BPM. Histograms showing the values of tested features in nonporated (empty bars) and porated (blue bars) locations. The purple and yellow solid lines show kernel smoothing density estimates. The title above each group of graphs indicates the dataset for which the histograms are shown.

BPM - depolarization - inner leaflet

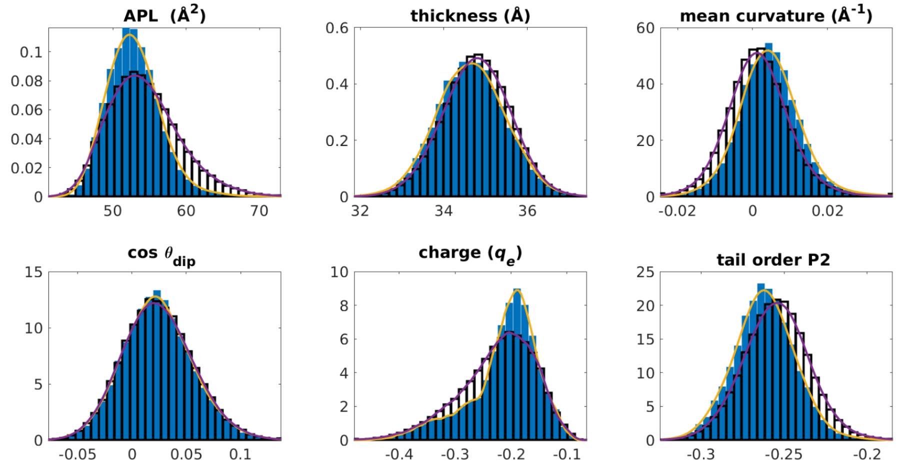

BPM - depolarization - outer leaflet

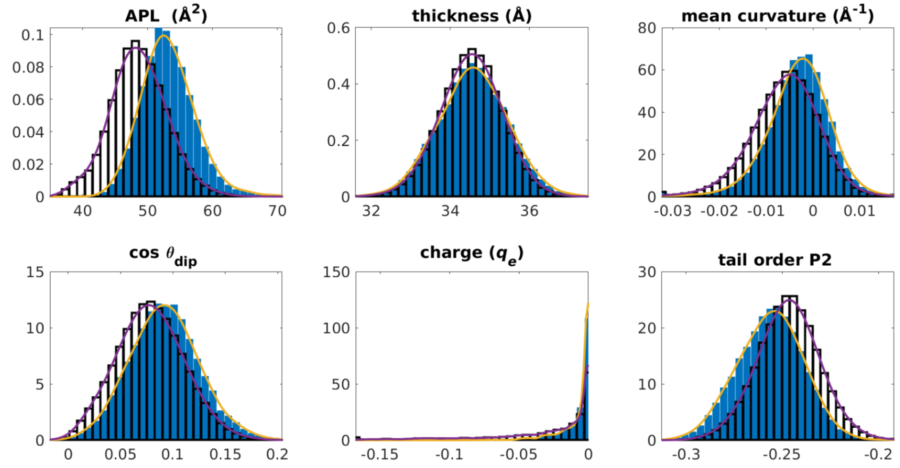

BPM - hyperpolarization - inner leaflet

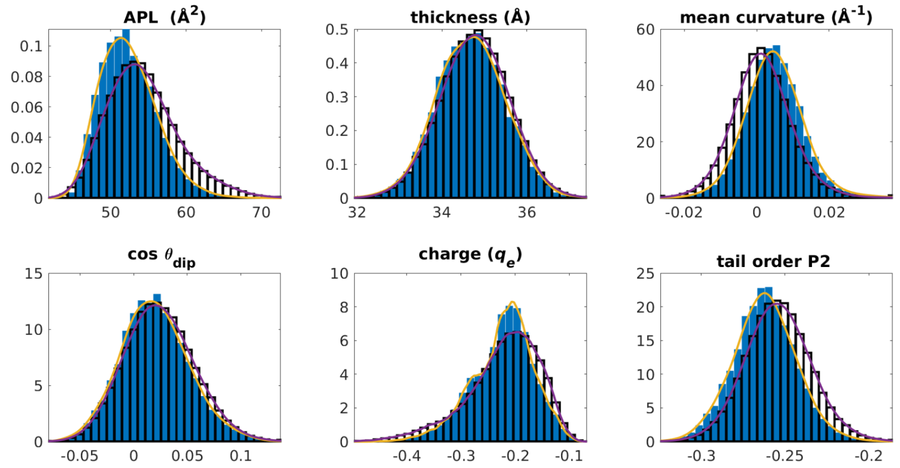

BPM - hyperpolarization - outer leaflet

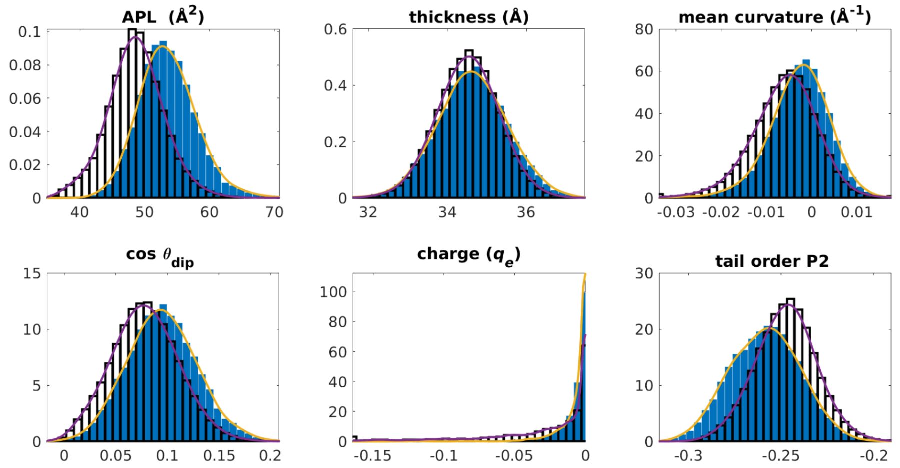

Histograms for BPM - continued.

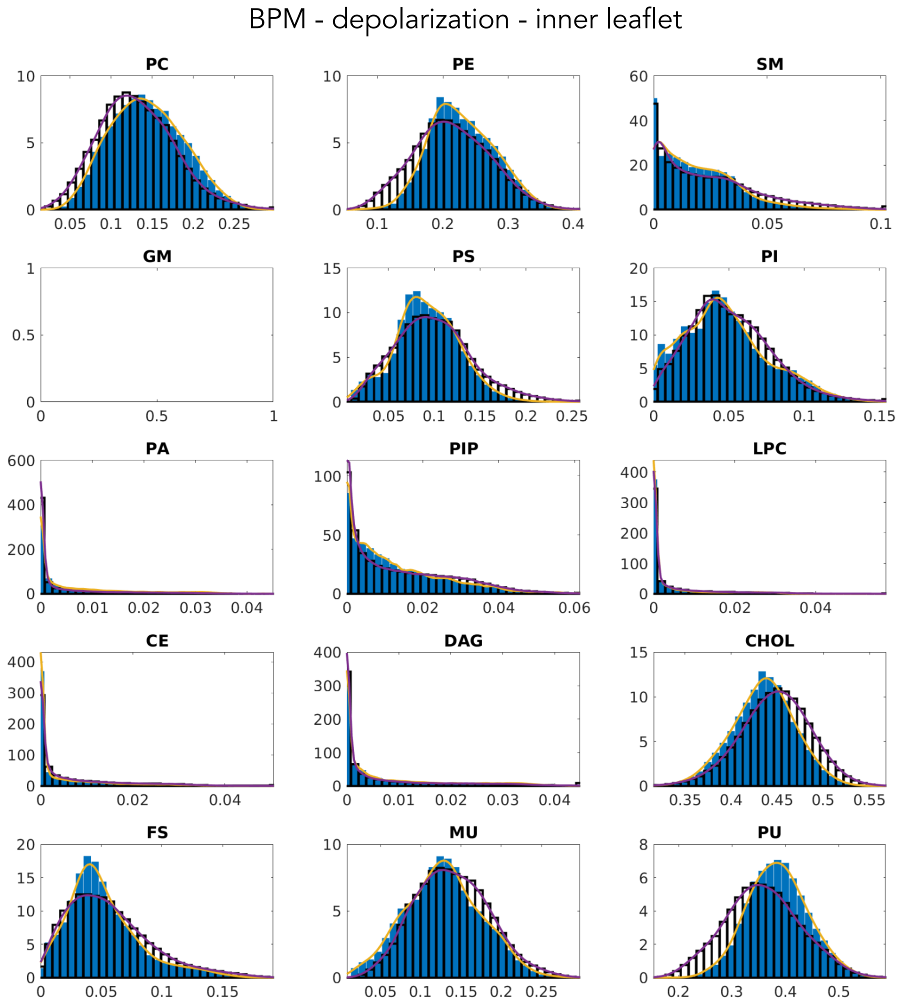

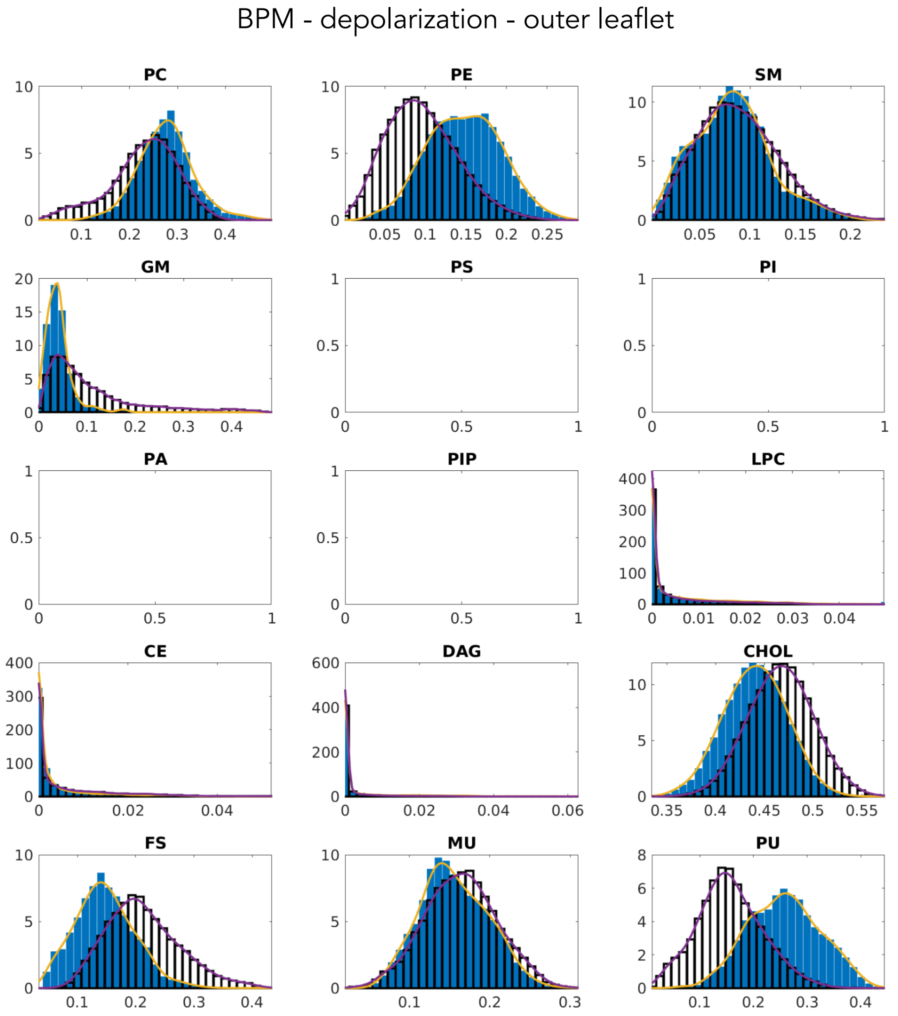

Histograms for BPM - continued.

BPM - hyperpolarization - inner leaflet

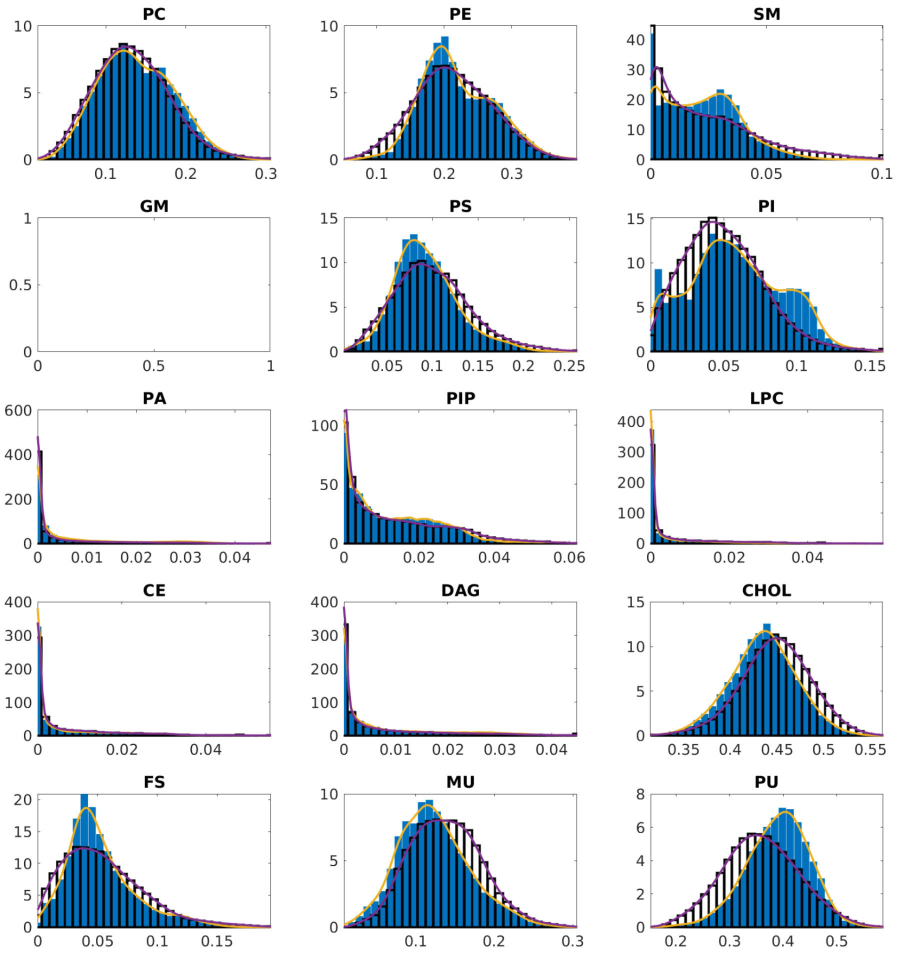

BPM - hyperpolarization - outer leaflet

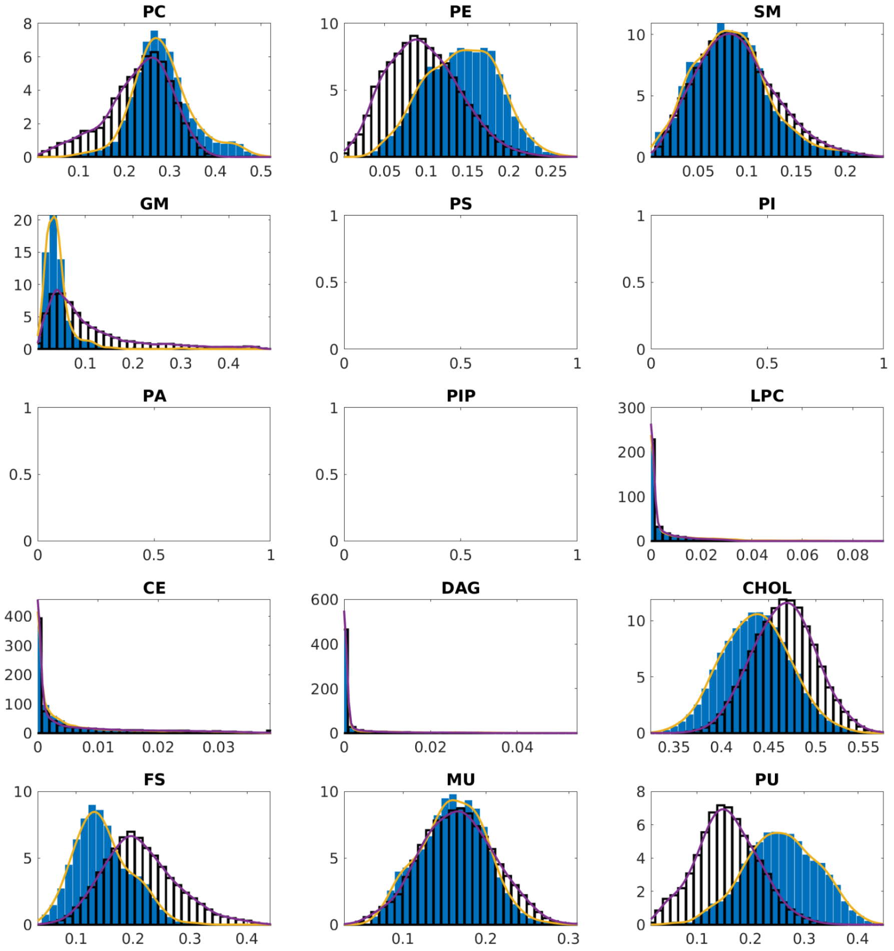

Supplement: Supplementary file 1. — Histograms showing the values of tested features in non-porated (empty bars) and porated (blue bars) locations, for average plasma membrane (APM) and for brain plasma membrane (BPM). The purple and yellow solid lines show kernel smoothing density estimates. The title above each group of graphs indicates the dataset for which the histograms are shown. [file elife-74773-supp1.pdf]
